# Update on Fundamental Mechanisms of Thyroid Cancer

**DOI:** 10.3389/fendo.2020.00102

**Published:** 2020-03-13

**Authors:** Alessandro Prete, Patricia Borges de Souza, Simona Censi, Marina Muzza, Nicole Nucci, Marialuisa Sponziello

**Affiliations:** ^1^Unit of Endocrinology, Department of Clinical and Experimental Medicine, University of Pisa, Pisa, Italy; ^2^Section of Endocrinology and Internal Medicine, Department of Medical Sciences, University of Ferrara, Ferrara, Italy; ^3^Endocrinology Unit, Department of Medicine (DIMED), University of Padova, Padova, Italy; ^4^Division of Endocrinology and Metabolism IRCCS Istituto Auxologico Italiano, Milan, Italy; ^5^Department of Medicine, University of Perugia, Perugia, Italy; ^6^Department of Translational and Precision Medicine, Sapienza University of Rome, Rome, Italy

**Keywords:** thyroid cancer, oncogenes, *RET*, *RAS*, *BRAF*

## Abstract

The incidence of thyroid cancer (TC) has increased worldwide over the past four decades. TC is divided into three main histological types: differentiated (papillary and follicular TC), undifferentiated (poorly differentiated and anaplastic TC), and medullary TC, arising from TC cells. This review discusses the molecular mechanisms associated to the pathogenesis of different types of TC and their clinical relevance. In the last years, progresses in the genetic characterization of TC have provided molecular markers for diagnosis, risk stratification, and treatment targets. Recently, papillary TC, the most frequent form of TC, has been reclassified into two molecular subtypes, named *BRAF*-like and *RAS*-like, associated to a different range of cancer risks. Similarly, the genetic characterization of follicular TC has been proposed to complement the new histopathological classification in order to estimate the prognosis. New analyses characterized a comprehensive molecular profile of medullary TC, raising the role of *RET* mutations. More recent evidences suggested that immune microenvironment associated to TC may play a critical role in tumor invasion, with potential immunotherapeutic implications in advanced and metastatic TC. Several types of ancillary approaches have been developed to improve the diagnostic value of fine needle aspiration biopsies in indeterminate thyroid nodules. Finally, liquid biopsy, as a non-invasive diagnostic tool for body fluid genotyping, brings a new prospective of disease and therapy monitoring. Despite all these novelties, much work remains to be done to fully understand the pathogenesis and biological behaviors of the different types of TC and to transfer this knowledge in clinical practice.

## Introduction

Thyroid cancer (TC) represents the most common endocrine malignancy, accounting for 3.4% of all cancers diagnosed annually (1). The transformation of thyroid follicular cells may result in differentiated or undifferentiated TC, through a multistep process that is the most accepted theory of follicular cell carcinogenesis (2). In this model, distinct molecular alterations have been associated with specific stages, driving progression from well-differentiated to undifferentiated follicular-derived thyroid carcinomas. More recently, the cancer stem-like cells theory has been proposed, according to which phenotypically different cancer cells could be generated by a small subpopulation of stem cells after genetic and epigenetic transformations (3). Differentiated TC, accounting for more than 90% of thyroid malignancies, comprises papillary thyroid carcinoma (PTC) and follicular thyroid carcinoma (FTC). Poorly differentiated thyroid carcinoma (PDTC) and anaplastic thyroid carcinoma (ATC) are rare tumors (5 and 1%, respectively) associated with aggressive behavior and short median time of survival (5 years and 6 months, respectively). Differently, medullary thyroid carcinoma (MTC), representing 5% of TC, arises from parafollicular C cells.

**Figure 1:**
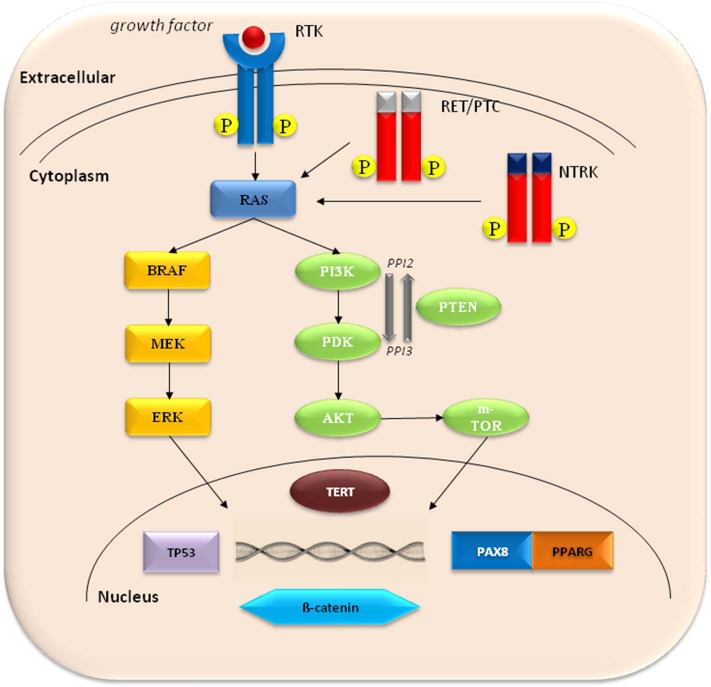
The molecular pathogenesis of thyroid cancer involves dysregulation of the mitogen-activated protein kinase (MAPK) and phosphatidylinositol-3 kinase (PI3K)/AKT pathways. Common activating mutations in the MAPK pathway include *RET*-PTC and NTRK rearrangements, and *RAS* and *BRAF* mutations. Common genetic alterations in the PI3K pathway include *RAS* mutations, *PTEN* mutations or deletions, *PIK3K* mutations or amplifications, and *AKT1* mutations. PAX8-PPARG fusions are common in FTC. Activation of Wnt/b-catenin pathway, inactivating mutations in TP53, and activating mutations in TERT promoter are frequent in undifferentiated thyroid cancer.

In the last 30 years, the availability of the genome sequence has produced much progress in elucidating the molecular mechanisms underlying TC (4). TC is a genetically simple disease with a relatively low somatic mutation burden in each tumor. Driver mutations, i.e., mutations that provide a selective growth advantage thus promoting cancer development, are identified in more than 90% of TC (4). The molecular pathogenesis of the majority of TC involves dysregulation of the mitogen-activated protein kinase (MAPK) and phosphatidylinositol-3 kinase (PI3K)/AKT signaling pathways. MAPK activation is considered to be crucial for PTC initiation, through point mutations of the *BRAF* and *RAS* genes or gene fusions of *RET/PTC* and *TRK*. On the other hand, PI3K/AKT activation is thought to be critical in FTC initiation and can be triggered by activating mutations in *RAS, PIK3CA*, and *AKT1* as well as by inactivation of *PTEN*, which negatively regulates this pathway. TC progression and dedifferentiation to PDTC and ATC involves a number of additional mutations affecting other cell signaling pathways, such as *p53* and *Wnt/*β*-catenin*. More recently, *TERT* promoter mutations have been described in all the histological TC type, with a significantly higher prevalence in aggressive and undifferentiated tumors, indicating their role in TC progression (Figure 1). Mutations in the *RET* (Rearranged during transfection) proto-oncogene account for most MTC cases and can occur sporadically or as inherited germline events in the multiple endocrine neoplasia type 2A (MEN2A) and 2B (MEN2B) syndromes. A minority of sporadic MTC are caused by *H-, K-*, and *N-RAS* mutations (Table 1).

**Table 1:** Distribution and frequency of known somatic mutations in different histotypes of thyroid cancer.

	**PTC**	**FTC**	**PDTC**	**ATC**	**MTC**
AKT	1% (5)	1-2.6% (6, 7)	–	0–3% (8, 9)	–
BRAF	61.7% (5)	1.7% (7)	19% (8)−33% (9)	19–45% (8–10)	–
DICER1	2.7% (5)	5.1% (6)	–	1.1% (9)	–
EIF1AX	1.5% (5)	5.1% (6)	10% (9)	9% (8)	0.6% (11)
HRAS	2% (5)	7% (7)	5% (9)	6% (8)	9.3–15.8% (11)
KRAS	1.26% (5)	4% (7)	2% (9)	0–5% (8, 9)	3.0–6.2% (11)
NRAS	6% (5)	17% (12)−57% (6)	21% (9)	18% (8)	0.6–1% (11)
PAX8-PPARγ	0.8% (5)	12% (13)−53% (14)	4% (9)	0 (8)	–
PI3KCA	–	5.5% (7)	2% (9)	18% (8)	–
PTEN	1% (5)	7.1% (7)	4% (9)	15% (8)	1% (11)
RET	–	–	–	–	55.8% (11)
RET/PTC	6.8% (5)	0 (7)	14% (9)	0 (8)	Very rare (15)
SWI/SNF	–	–	6% (9)	18–36% (8, 9)	–
TERT promoter	9.4% (5)	–	33–40% (8, 9)	43–73% (8–10)	–
TP53	6% (5)	5.1–9.7% (6, 7)	0–8% (8, 9)	43–78% (8–10)	1.2% (11)
TSHR	2% (5)	10.3% (7)	2% (9)	6% (8)	0.6% (11)

## Papillary Thyroid Carcinoma

The majority of PTC has an excellent prognosis in terms of long-term survival (>90%) (4, 16), although recurrent disease rates reported are rather high, occurring in 25–35% of patients (4, 17, 18). The clinical challenge relies in the early identification of those patients who need aggressive treatment from the beginning from those who will have an indolent course.

At the molecular level, several driver mutations have been associated with PTC malignancy and clinicopathological features, but none has proven useful in directing treatment and determining clinical outcome. Among these, *RET* rearrangements or point mutations of *RAS* or *BRAF* proto-oncogenes have been described and are found in an almost mutually exclusive modality in nearly 70% of PTC (Table 1). These genetic alterations are common in PTC, leading to (constitutively) activation of MAPK or PI3K signaling pathways (19).

RET proto-oncogene encodes for a tyrosine kinase receptor and its activation invokes intracellular signaling cascades, leading to gene expression modulation and biological responses. RET/PTC fusion protein maintains the tyrosine kinase domain intact and enables uncontrolled activation of the MAPK signaling cascade (20). *RET* rearrangement was first reported by Fusco et al. (21), and in the following years, different types of *RET/PTC* rearrangements have been identified (15). *RET/PTC1* and *RET/PTC3* are the most common (5), the latter being frequent in post-Chernobyl children due to radiation exposure. The prevalence of RET rearrangements in PTC has varied deeply among studies (2.5–73%) (22, 23) probably due to ethnical and geographical variations as well as to the method used for their identification and genetic heterogeneity, as demonstrated by Zhu et al. (24); recent reports, however, from the Tumor Cancer Genome Atlas (TCGA) in a series of 484 PTC belonging to different ethnic groups, only 6.8% presented RET rearrangements (5). Some reports have indicated *RET/PTC1* as being associated with a more favorable prognosis, while *RET/PTC3* was associated with a more aggressive and malignant phenotype (25, 26). However, patients harboring these rearrangements usually follow a favorable course, owing to their ability to respond well to radioactive iodine (RAI) therapy (27). It is of interest that in post-Chernobyl TC, other rearrangements have been found: in particular, *TRK* gene and *BRAF* gene fusions (28). Recently, *NTRK* fusions have been reported in some series of advanced cancers and have been proposed as novel targets of cancer therapy (29).

BRAF, a member of the raf family of serine/threonine protein kinases, has been shown to be mutated and constitutively activated in ~7% of all cancers. Prevalence of BRAF mutation in PTC varies among different series ranging from 29 to 83% (30–37). Most recently, the TCGA reported 74.6% of BRAF mutations in PTC, of which 61.7% were V600E substitutions. Different genetic alterations have been identified in this gene; however, the majority of classic PTC (cPTC) harbor the *BRAF*^*V*600*E*^ variant (32). The mutation of *BRAF* promotes the activation of downstream transcription factors, leading to cell differentiation, proliferation, growth, and apoptosis. Several studies reported an association between the *V600E* variant and aggressive disease features, including lymph node metastases, invasion, and recurrence (38, 39). Intratumor genetic heterogeneity involving *BRAF* mutation has been demonstrated and the clonal/subclonal status of *BRAF*^*V*600*E*^ could account for the conflicting results on the prognostic value of this variant (5, 40, 41), as well as may explain the lack of complete response to targeted therapies (42).

RAS is a family of GTP-binding proteins, upstream of BRAF, that acts through the MAPK and PI3K-AKT signaling pathways. *HRAS, KRAS*, and *NRAS* encode for four different but related proteins (H-Ras, N-Ras, K-Ras4A, and K-Ras4B) that are cardinal in controlling cell growth, differentiation, and survival. Missense mutations at codons 12, 13, and 61 lead to constitutive activation of RAS signaling, which is found mutated in >30% of all tumors, including thyroid lesions both benign and malignant (43). In fact, *RAS* mutations may be found in FTC (28–68%), in follicular-variant PTC [FVPTC; up to 43% (31)], and in its non-invasive FVPTC [NIFTP; up to 47% (44)], as well as in follicular adenomas (20–25%) (45), showing the limited role for *RAS* mutations alone in the clinical outcomes of TC (5, 46).

TERT encodes for the telomerase reverse transcriptase, and two hotspot genetic alterations have been reported (C228T and C250T). These mutations promote telomerase activity and telomere length maintenance in cancer cells and are present in nearly 10% of PTC (Table 1). There are consistent data linking them to PTC aggressiveness when in co-presence of a driver mutation, indicating a possible role for TERT mutations in PTC progression and prognosis (47).

Among PTC variants, NIFTP represents a novel entity with an almost negligible risk of negative outcome (46). Many efforts have been produced in order to identify a unique NIFTP genomic profile that helps to diagnose these tumors by FNAB. A recent article showed that the NIFTP genomic profile is more similar to FTC than PTC. Interestingly, 67% of NIFTP harbored *RAS* mutations alone or in tandem with other mutations (*p53* and *PTEN* mutations), whereas *BRAFV600E* mutation was not described. Furthermore, 22% of NIFTP presented *PAX8/PPARG* and *THADA/IGF2BP3* gene fusion mutations (48). However, although NIFTP genomic profile seems to be different from those of other PTC variants, the degree of overlap makes it difficult to identify NIFTP with FNAB (49) and molecular analysis.

Recently, the TCGA has unfolded the genomic landscape of TC, reducing to <4% the unknown genomics of PTC (5). Based on a *BRAFV600E-RAS* gene expression score, PTCs may be grouped according to their molecular differences as *BRAFV600E*-like and *RAS*-like PTC. In fact, *BRAFV600E* mutation is more frequent in cPTC and tall-cell variant PTC, showing increased MAPK activation, whereas *RAS* mutations occur mostly in FVPTC and NIFTP, having a genomic profile more similar to FTC. The genomic landscape described by these studies reveals that PTC bears a relatively stable genome, which could explain the usually indolent course of this disease. Nonetheless, aggressive PTC may occur and, therefore, additional investigation is necessary in order to early identify those PTCs that will dedifferentiate and become life-threatening.

## Hyalinizing Trabecular Tumor

Hyalinizing trabecular tumor (HTT) is a rare benign follicular neoplasm characterized by thick trabeculae and cells with nuclear elements shared with PTC, producing false-positive cytology (50). However, using whole-exome and RNA-Seq analyses, Nikiforova et al. presented a unique genomic signature of HTT and showed that GLIS fusions, especially PAX8-GLIS3, are highly prevalent in HTT but not in PTC. These fusions were related to overexpression of GLIS, inducing upregulation of extracellular deposition of collagen IV (51).

## Follicular Thyroid Carcinoma

In 2017, the World Health Organization guidelines proposed to re-classify the FTC into minimally invasive (miFTC), encapsulated angioinvasive (eaFTC), and widely invasive (wiFTC) subtypes, according to their different clinical and biological behaviors (52). Although the genomic landscape of PTC is nearly complete, the molecular characterization of FTC and its progression from miFTC to wiFTC are still not totally clear.

In FTC, the most common mutations are in the *RAS* gene family (HRAS, KRAS, and NRAS), and *NRAS* gene was found mutated in 17% (12) to 57% (6) of cases. Although a previous study had demonstrated that *RAS* mutations are negative prognostic markers (53), recent evidences did not describe RAS mutations as predictors of disease-specific mortality (54). Intriguingly, *RAS* mutations appear to be mutually exclusive with *TSH* receptor mutations, which were found in 10.3% of FTC cases (54) (Table 1).

The fusion gene *PAX8-PPAR*γ was identified in one-third of FTC cases, ranging from 12% (13) to 53% (14) (Table 1). *PAX8* is a member of paired box family of transcription factors, and it is necessary for the physiological thyroid development, promoting thyroid progenitor survival and driving the expression of thyroid-specific genes (55). Conversely, *PPAR*γ is a member of the nuclear receptor family of transcription factors, and, besides its role of master of adipogenesis, it seems to be a tumor suppressor gene (56). The fusion protein PAX8-PPARγ can act as a negative inhibitor of oncosuppressor PPARγ activity or as a novel transcriptional factor with protooncogene activity (57). However, it seems to not affect FTC prognosis (13).

TERT promoter mutations have been described in about 15% of FTCs (Table 1) and associated with worst clinical and prognostic features (58). Furthermore, many groups (54, 59, 60) described point mutations of driver genes *EIF1AX* and *DICER1* and somatic arm-level copy changes (e.g., loss of 22q), the significance of which needs to be clarified.

In this complex scenario, the total mutational burden seems to be a prognostic factor: the bigger is the number of mutations, the worse is the prognosis. Furthermore, since multivariate analysis describes the total mutational burden as an independent indicator of histopathology, the genetic analysis may be used to predict survival as a complement to the histological informations (54).

## Poorly Differentiated Thyroid Cancer

The frequency of molecular mutations in PDTC is not the same in all studies, and this can be related not only to the sensitivity of the molecular technique used to ascertain the molecular pattern (Next-generation sequencing vs. Sanger sequencing) but also to the histological criteria used to define the PDTC. Indeed, two main classifications for PDTC exist: Turin (the presence of a solid/trabecular/insular pattern of growth, in the absence of the conventional nuclear features of PTC and at least one of the following: convoluted nuclei, high mitotic rate, or tumor necrosis) and MSKCC criteria (high mitotic rate and necrosis independently from the growth pattern) (8, 61–63). *BRAF* mutations are found in 19% (8)−33% (61) of PDTC, while *H*-, *K****-***, and *N****-****RAS* mutations were found in 5% (61)−28% (8) of cases (Table 1). *BRAFV600E* was found to be more frequent in PDTC when defined following the MSKCC criteria, while *RAS* mutations are more common in PDTC fulfilling Turin definition (8). Moreover, *BRA*F and *RAS* are mutually exclusive and correlate with a different clinical behavior: *BRAF*-mutated PDTCs were found to have a higher rate of nodal metastases vs. a higher rate of distant metastases found in *RAS*-mutated PDTCs. Furthermore, the expression of thyroid-specific genes related to radioiodine avidity was found to be lowered in *BRAF-*mutated PDTCs, but not in their *RAS* mutated counterparts (8).

*TERT* promoter mutation can co-occur with *BRAF* and *RAS* mutations (8)and is particularly frequent in advanced tumors: 33% (61)−40% (8) of PDTCs are found to carry a *TERT* promoter mutation (Table 1), inducing a higher risk of distant metastases and mortality (8). Intriguingly, *TERT* promoter mutation in PTC is subclonal, while it is clonal in advanced cancers (PDTC and ATC), suggesting an advancement in TC due to a selected immortalized *TERT*-positive clone (8). By contrast, p53 was rarely found in PDTC, even using sensible techniques, having a frequency of 8% in the Landa et al. analysis, with no patients carrying *p53* mutation in PDTC in the Elisei et al. series (8, 61). *EIF1AX* mutations are present in 1% of PTC and in 10% of PDTC, inducing a worse survival (8) (Table 1). Interestingly, in advanced cancers, it has a strong association with *RAS* mutations, while in PTC, these two mutations are mutually exclusive. The significance of this observation is yet to be clarified. *EIF1AX* mutation does not overlap with *PI3K/AKT/mTOR* pathway mutations, suggesting similar functions in thyroid progression. Also, *PTEN/PI3KCA* is uncommon (8) or even absent in PDTC (61).

Another substantial difference in thyroid advanced tumors compared to PTC is in the chromosome number variation. The genome of PTC is largely diploid, while in PDTC and ATC, chromosome copy number alterations are widespread and more frequent in those tumors lacking a driver gene mutation (8). Gene rearrangements common in PTC (*RET/PTC, PAX8-PPAR*γ, *ALK* fusions) may be found in 14% of PDTC (specially in younger patients), but are absent in ATC (8).

## Anaplastic Thyroid Cancer

In ATC, *BRAF* and *H-, K-*, and *N-RAS* mutations have a frequency of 19–45% and 9.5–27%, respectively (8, 9, 61), lower than that of DTC. Conversely, the two most frequent mutations occurring in ATC are *TERT* promoter mutations, occurring in 43–73% of cases and *TP53* mutations that have a frequency ranging from 48 to 73% of cases (8, 9, 61). Interestingly, while *TERT* promoter mutations are quite common also in PDTC, *TP53* is highly frequent only in ATC and thus it may be considered pathognomonic for this tumor and its severe aggressiveness (8). Also, mutations in *PTEN* and *PI3KCA* are rather frequent in ATC, 15 and 18%, respectively, in comparison with well-differentiated cancers and PDTC (8). Moreover, in ATC, other mutations less typical for thyroid tumors are also found: 18–36% of ATC carry mutations in *SWI/SNF* chromatin remodeling complex (8, 9) and in genes associated with histone modifications (8) (Table 1); mutations in genes involved in cell-cycle regulation (*CDKN2A, CDKN2B*, and *CCNE1*) are present in 29% of ATC (8), and finally, few ATCs were also found to be mutated in tumor immune regulation genes (*PDL1, PDL2*, and *JAK2*) (8). The real pathogenic role of these alterations is unknown and could be related to the genomic instability of these tumors.

Interestingly, in this panorama of apparently heterogeneous molecular scenario, four distinct subtypes of molecular pattern of ATC have been proposed: (1) type 1 ATC, *BRAF*-positive ATC, with a genetic landscape similar to PTC (it is likely to evolve from PTC); (2) type 2 ATC, *NRAS*-positive ATC, which may originate from FTC; (3) type 3 ATC, which carries *RAS* mutations or more atypical ones (e.g., *PTEN, NF1* and *RB1*) and is likely to originate from FTC or from Hürthle cell carcinoma; and (4) mixed ATC, which harbor loss-of-function genetic alterations and mutations in the genes of cell-cycle regulations (*CDKN2A* and *CDKN2B*) (9) and do not seem to derive from a pre-existing DTC.

Intriguingly, ATC presents a deeper status of dedifferentiation than DTC and PDTC: in ATC, mRNA levels for *TG, TSHR, TPO, PAX8, SLC26A4, DIO1*, and *DUOX2* genes are profoundly supressed (8). In *BRAF*-positive ATC, *TP53* or *PIK3CA* mutations are frequently found and may drive the dedifferentiation process. Among ATC tumors carrying *RAS* mutations, the mechanism of dedifferentiation is less clear, although *EIF1AX* is a good candidate, given its frequent co-occurrence with *RAS* mutations in advanced cancers (10). Among ATC tumors not carrying *BRAF* or *RAS* mutations, the atypical mutations of *NF1, ERBB2, mTOR*, and *MHL* genes may enhance the dedifferentiation process (10).

## Medullary Thyroid Cancer

MTC can be either familial (25%) or sporadic (75%), and in both cases, proto-oncogene *RET* exerts a crucial role in its oncogenesis. Virtually, all familial cases (>98%) present germline *RET* mutations (64). However, two cases of familial MTC without any *RET* germline mutations have been recently described with one case carrying a germline mutation of *ESR2* gene (65) and another one of MET gene (66). In sporadic cases, *RET* is mutated in 44% and *RAS* genes (mainly *HRAS* and *KRAS*) are mutated in 13% of cases, according to COSMIC (catalog of somatic mutations in cancer) database (7). Intriguingly, oncogenesis of a relevant group of sporadic (more than 40%) and some rare familial MTCs is still unclear.

In MTC, *RET* gene is typically harboring point mutations, while its deletions or insertions are rare. Activating point mutations of *RET* may affect both extracellular and intracellular domains, inducing different effects: intracellular mutations domain induce a ligand-independent constitutive dimerization, promoting the activation of the tyrosine kinase receptor; otherwise, extracellular mutation domains induce a ret activation, which is ligand and dimerization independent (15). Mutations of different ret domains induce different clinical features in familial MTC cases. They are grouped into MEN2A and MEN2B syndromes, and the MEN2A syndrome is subdivided by clinical characteristics into MEN2A associated with cutaneous lichen amyloidosis (CLA), MEN2A with Hirschsprung Disease (HD), and familial MTC (FMTC) (67). There is an evident genotype–phenotype correlation with patients affected by classical MEN2A harboring almost exclusively *RET* codon 634 mutations (68), patients affected by MEN2A and HD carrying *RET* germline “Janus” mutations in exon 10 (codons 609, 611, 618 and 620) (69), and patients with MEN2B harboring almost exclusively *RET* germline mutations in exon 16 (codon M918T) (70).

The genetic profile in sporadic MTCs is more heterogeneous than in familial MTCs. Recently, the genetic landscape of 208 cases of sporadic MTCs, identified by using a deep sequencing technique, has been published (11). In this large series, the number of *RET* or *RAS* negative cases was highly reduced (18.3%), and the crucial pathogenic role of *RET* and *RAS* gene, which are affected by mutually exclusive alterations, has been confirmed. According to these data, *RET* mutations remain the most common genetic variant in sporadic MTCs (55.8%) followed by *RAS* mutations (24.3%) (Table 1). Interestingly, the study also demonstrated that patients with *RET*-positive MTCs have a lower survival than those with *RAS* mutations. Moreover, the variant allele frequency represents an additional prognostic marker in *RET*-positive MTCs (11).

## Immune Profile of TC

Increasing evidences confirm that solid tumors are composed by different clusters of cells including cancer cells, cancer stem cells, fibroblasts, and stroma cells, and also a variety of cells belonging to the innate and adaptive immune system (71). Nowadays, the evidence that tumor cells and immune cells have an important relationship inside tumor microenvironment is recognized worldwide. Furthermore, the presence in some solid tumors of an intensive infiltration and the evidence of a contemporary anti-tumor response and a tolerant microenvironment, essential for tumor growth and progression, enforce the role of the immune system inside cancer (72). In this context, a comprehensive study of the immune profile of TC to clarify the mechanisms involved in immune escape and characterize the tumor microenvironment results is pivotal.

Over the last years, many studies have focused on cancer gene expression profiling, outlying a detailed immune profile also for TC. In 2018, a classification arisen from a huge research on the TCGA databases detected six immune subtypes of cancers (73): C1—Wound healing; C2—IFN-γ dominant; C3—Inflammatory; C4—Lymphocyte depleted; C5—Immunologically quiet; C6—TGF-β dominant. Following this classification, the majority of PTCs were classified as C3 tumors, with a balance in T helper1:T helper2 presence, elevated T helper 17 genes, low tumor cell proliferation, and lower levels of aneuploidy and somatic copy number alterations. This was in agreement with the former observation of a complex immune network inside PTCs consisting of a rich infiltration by tumor-associated macrophages, myeloid-derived suppressor cells, and T helper 17 cells (74).

More recently, the development of an immunoscore stratification, based on immune contexture within the tumor, has allowed the classification of cancers by their immune phenotype. Indeed, thanks to the distribution of T CD3+ and T CD8+ lymphocytes in the center of the cancer or at the invasive margin, it could be possible to distinguish four different phenotypes of cancers: (1) the hot ones, with a high infiltration of cells all over the tumor; (2) altered–excluded with the presence of cells only at the invasive margin; (3) altered–immunosuppressed with sparse immune cells within all the tumor; and (4) cold tumors, without infiltration (75). A study published in 2019 analyzed a cluster of about 730 immune-related genes in the three major histotypes of TC (i.e., PTC, PDTC, and ATC) with the aim to investigate and clarify the immune profiling of advanced TCs (76). The histotypes are segregated into two different clusters of expression: a first group, including PDTCs, part of PTCs, and normal thyroid, and a second group including ATCs and part of PTCs. Interestingly, the regulation of gene expression was different between ATCs and PDTCs: the first ones had a marked overexpression of about all immune genes analyzed, compared to normal tissue; the second ones had expression levels that were very similar to normal thyroid. The results obtained indicated the existence of two major immune phenotypes in TCs: an ATC-like one, including hot and altered–immunosuppressed tumors, and a PDTC-like one, including altered–excluded and cold tumors. Moreover, TCs, mostly the anaplastic ones, showed an increased overexpression of immune checkpoints, including PDL1, PDL2, PD1, LAG-3, TIM-3, PVR, and TIGIT. These data confirm a strong activation of adaptive immune escape strategies for blocking tumor-infiltrating leucocytes, especially in ATC (77).

## Current and Future Clinical Applications

Significant advances in the understanding of TC biology, coupled with advances in high-throughput technologies, are contributing to the development of novel diagnostic, prognostic, predictive, and therapeutic tools for TC patients.

Most efforts have been made in the development of molecular tests for cancer diagnosis in thyroid nodules. Panels of gene expression markers [e.g., Afirma Genomic Sequencing Classifier (78)] or somatic mutation panels [e.g., ThyroSeq Genomic Classifier (79)] have improved the pre-operative diagnostic accuracy for patients with indeterminate cytology by addressing the problem of unnecessary surgery for benign thyroid nodules. Much effort should be done in order to pre-operatively identify a subset of aggressive cancers or to increase positive predictive value in some tumor subtypes (i.e., in *RAS*-mutated cases).

An alternative approach for early diagnosis and prompt detection of disease persistence or relapse is liquid biopsy (i.e., the sampling and non-invasive analysis of circulating tumor-derived material, the *tumor circulome*) (80), and its details are reported elsewhere (81). Although the development of circulating biomarkers for TC is still in its infancy and at present liquid biopsy does not find any application, it presents several advantages, such as the rapid, low-cost, non-invasive nature of sample collection and the capture of intratumoral and intermetastatic genetic heterogeneity. The diagnostic application of circulating tumor DNA (ctDNA) in follicular cell-derived TC is still questioned. In contrast with other advanced cancers, only 25% of metastatic TCs have detectable ctDNA (82). These data are confirmed by multiple studies focusing on detection of *BRAFV600E* mutation in PTCs, which showed low or no concordance between plasma and tissue samples (83), also when sensitive techniques were employed (84). Conversely, higher concordance was found in ATC (85) and MTC (86) patients with important implications in guiding treatment selection and clinical trial enrollment.

Circulating miRNAs represent an alternative and valuable source for real-time thyroid tumor monitoring, due to their high stability in biological fluids (87) and tissue specificity (88). Most studies published thus far have been conducted on PTC patients, and in this setting, unlike ctDNAs, circulating miRNAs show undeniable promise as novel diagnostic and predictive biomarkers. Higher circulating levels of miR-221-3p, miR-222-3p, and miR-146b-5p were detected in PTC patients than in healthy controls, while miR-222 and miR-146b levels also discriminate between PTCs and benign nodules. Moreover, circulating levels of miR-146b-5p, miR-221-3p, miR-222-3p, and miR-146a-5p have been shown to decline after tumor excision (89). Recently, miRNAs of tumor tissue have been proposed to face the challenge of indeterminate FNAB category. Stokowy et al. showed that none of the miRNAs could be used as an alone malignancy marker but the classifier made by miR-484/miR-148b-3p identified TC with a sensitivity of 89% and a specificity of 87% (90).

Furthermore, miR-221-3p and miR-146a-5p blood levels in PTC patients have been shown to predict clinical responses, with significantly increased levels observed at the 2 year follow-up in patients with structural evidence of disease, including some in which serum thyroglobulin assays remained persistently negative (91).

Realization of this enormous potential will depend on our ability to develop standardized methods for detection of circulating biomarkers and to validate their performance in clinical setting.

## Conclusions

We discussed the molecular mechanisms involved into the pathogenesis of the different types of TC and their clinical relevance. In the last years, many steps forward have been made in the genetic characterization of TC, providing molecular markers for diagnosis, risk stratification, and treatment targets. However, many other steps need to be done in order to diagnose TCs with aggressive behavior, to tailor the most appropriate target therapy, and to monitor the response to the therapies using new molecular approaches.

## Author Contributions

All authors listed have made a substantial, direct and intellectual contribution to the work, and approved it for publication.

### Conflict of Interest

The authors declare that the research was conducted in the absence of any commercial or financial relationships that could be construed as a potential conflict of interest. The handling editor declared a past co-authorship with the author MM.
